# Nuclear energy in Bangladesh: A SWOT analysis

**DOI:** 10.1016/j.heliyon.2024.e31933

**Published:** 2024-05-24

**Authors:** Faiyaz Fahim, Abdulla Al Farabi, Md Sabid Hasan, S.M. Naimur Rhaman Sayam

**Affiliations:** a Institute of Nuclear Power Engineering, Bangladesh University of Engineering and Technology, Dhaka, Bangladesh; b Department of Electrical and Electronic Engineering, Bangladesh Army University of Engineering and Technology, Natore, Bangladesh

**Keywords:** Nuclear Energy, Rooppur Nuclear Power Plant, SWOT, Energy Policy

## Abstract

Zero-emission energy sources like nuclear energy are taken into account worldwide due to the negative environmental consequences of fossil fuels and their limited availability over the years. Bangladesh plans to add nuclear energy to its portfolio of energy sources by 2024. Under the scope of this study, the country's nuclear energy strategy was looked at in terms of its strengths, weaknesses, opportunities, and threats. This was done using a method known as SWOT analysis. It has come to light that Bangladesh has a significant number of aspects in terms of strengths as well as opportunities that make the possibility of doing an investment in the construction of a nuclear power plant a realistic choice. Difficulties such as a culture of poor maintenance, financial burden, lack of skilled manpower, a poor power grid, radioactive waste management, and corruption are the weaknesses and threats to the effective construction, operation, and maintenance activities of the nuclear power plant in Bangladesh. By mitigating these difficult aspects, Bangladesh's journey to adopt nuclear energy can be more smooth.

## 1 **Introduction**

In this contemporary age, ensuring a steady supply of energy is crucial to any country's ability to advance economically and increase its citizens' standard of living. An increase in both population and technical sophistication are major contributors to the dramatic rise in worldwide demand for energy. The demand for and consumption of primary energy in Bangladesh has seen a significant spike over the last three decades as a direct result of the country's relatively higher rate of macroeconomic development as well as favorable policy reforms in trade and investment [1]. When viewed from this vantage point, having reliable access to electricity is one of the most important factors in promoting economic growth, development, and social welfare.

Nuclear power plants generate electricity through the fission chain reaction. Fission chain reactions are made up of a series of nuclear fission, which is the splitting of atomic nuclei. Each fission is set off by a neutron made by the previous fission. At present, nuclear power plants contribute to 10 percent of worldwide generation of electricity. In many countries throughout the globe, electricity facilities that rely on fossil fuels have the potential to be replaced by nuclear power plants. The shift away from traditional forms of electricity generation and toward nuclear energy is anticipated to alleviate demand pressures on fossil fuels while simultaneously shielding the environment from the effects of emissions of greenhouse gases [2]. Furthermore, the integration of nuclear energy has the potential to enhance the sustainability of the supply chain within the industrial sector of Bangladesh by diminishing the dependency on fossil fuels and ensuring consistent energy sources [3].

In order to ensure long-term access to electricity that is both safe and affordable, the government of the People's Republic of Bangladesh has placed a priority on using a wide variety of fuel sources and incorporating cutting-edge technology. The country has made the decision to construct and operate a nuclear power plant in order to satisfy its growing demand for electricity that is dependable, affordable, and environmentally friendly; to cut down its reliance on fossil fuels to generate electricity; and to diversify its portfolio of energy sources.

Energy diversity plays a crucial role in propelling the advancement of macroeconomic development. According to a comprehensive study focusing on the member countries of the Asia-Pacific Economic Cooperation (APEC), it has been revealed that the act of diversifying energy sources plays a pivotal role in promoting sustainable development. This correlation between energy source diversification and sustainable development has been deemed more substantial than factors such as Information and Communication Technology (ICT) or economic globalization, although it was found that social globalization tends to have an adverse impact. The outcomes of this research underscore the significance of energy diversity in the pursuit of sustainable development within the region [4]. In a study conducted by Shahbaz et al., an in-depth analysis was carried out to explore how financial development and various other determinants influence the diversification of energy sources within Australia. Their results unveiled a distinct curvilinear relationship between financial development and energy diversification, showcasing a pattern that follows an inverted-U trajectory [5]. Furthermore, Ahmed et al. delved into investigating the association between energy diversification and economic growth in Nordic countries. Their study uncovered a lasting positive influence on economic growth as a result of energy diversification efforts. Despite acknowledging the potential short-term financial drawbacks associated with energy diversification initiatives, their research highlights a prospective upside that could be attained in the long run [6].

The primary objective of this research initiative is to thoroughly evaluate and determine the viability and practicality of integrating nuclear energy as a component of Bangladesh's portfolio of energy sources. This was achieved by using a comprehensive SWOT analysis, which entails evaluating the strengths, weaknesses, opportunities, and threats associated with the construction of a nuclear power plant in Bangladesh.

## 2 Bangladesh: at a glance

Bangladesh is located in the eastern portion of the Indian subcontinent. It sits between 20°34′ and 26°38′ north latitude and 88°01′ and 92°41’ east longitude. The country is surrounded on the west, north, and east by India, and on the south by the Bay of Bengal and a tiny border strip with Myanmar. It has a land area of 147,570 square kilometers. It is divided into seven divisions: Dhaka, Chittagong, Khulna, Sylhet, Rajshahi, Barisal, and Rangpur [7]. On the bank of the Padma River, in the Ishwardi upazila of the Pabna district, is where the Rooppur Nuclear Power Plant is currently being constructed. It is in the northwest of Bangladesh, between 140 and 160 km west of Dhaka, the capital city.

## 3 Power system master plan 2016 and nuclear energy

**Fig. 1:**
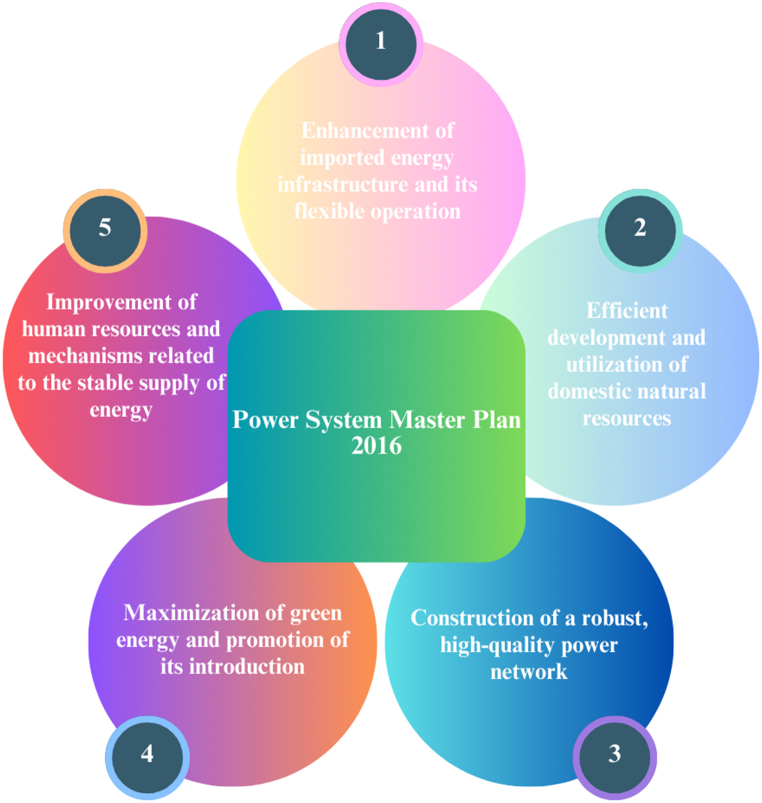
The key points of Power System Master Plan 2016.

The Power System Master Plan 2016 (PSMP 2016) was prepared with the support of one of Bangladesh's development partners, the 10.13039/501100004532Japan International Cooperation Agency (10.13039/501100004532JICA). In order to achieve Bangladesh's goal of becoming a high-income economy by 2041, the plan intends to assist Bangladesh in developing a comprehensive energy and power development strategy. The execution of the master plan should be guided by the five key perspectives of all stakeholders involved in reaching this goal [8]. Additionally, it includes an economic expansion strategy, a primary energy balance plan, a power balance plan based on the power development plan, and an energy pricing policy. Fig. 1 illustrates five main master plan perspectives.

**Fig. 2:**
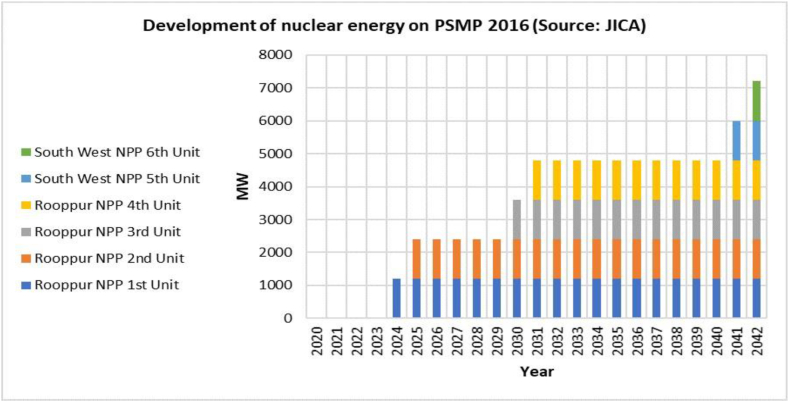
Development of nuclear energy on PSMP 2016 [8].

The load curve, also known as the daily electricity demand, exhibits variations based on factors such as season, temperature, and time of day. Due to the inherent inability to store electricity, it is imperative that it be promptly consumed. Nuclear power plants play an important role in maintaining a stable base load. Following discussions with key organizations, Fig. 2 depicts the PSMP 2016 scenario for nuclear power plants. According to PSMP 2016, the first unit (1200 MW) is expected to begin operations in 2024, and the second (1200 MW) in 2025. Energy development planning incorporates these values without alternatives. Therefore, nuclear energy is assumed to be one of the fixed components of generation capacity in the simulation. This is done by considering the government's nuclear power plant project planning [8].

## 4 The present and future aspects of Bangladesh’s electricity generation

In this section, the present and future of Bangladesh's electricity generation mix have been discussed.

### 4.1 Current electricity generation scenario

**Fig. 3:**
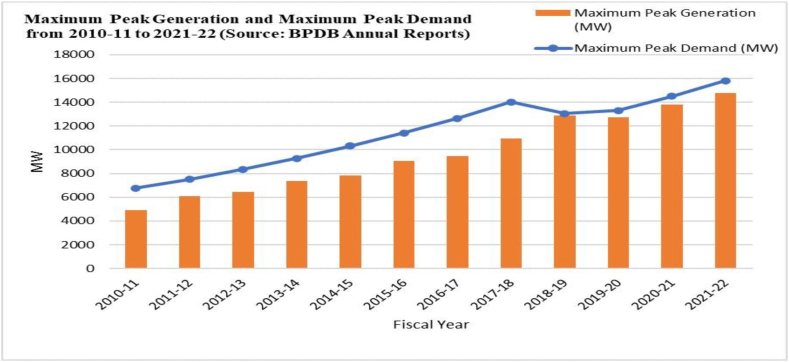
Maximum peak generation and maximum peak demand from 2010-11 to 2021-22 (Source: BPDB Annual Reports).

**Fig. 4:**
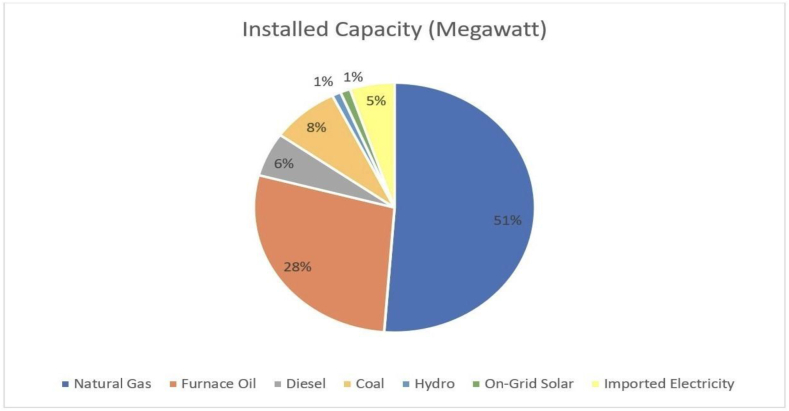
The breakdown of Bangladesh's installed capacity by source [9].

Currently, Bangladesh has an installed capacity of 22,608 MW, which barely fulfills the country's ever-increasing electricity demand. Fig. 3 depicts the widening disparity between electricity demand and generation as a result of reliance on traditional energy sources. Natural gas is the most important source of energy in Bangladesh, according to the Bangladesh Power Development Board, which places furnace oil and coal in second and third place, respectively [9]. However, according to Petrobangla data, Bangladesh's current natural gas reserves are 11 Trillion cubic feet (Tcf). Local natural gas supply has dropped from 0.969 Tcf in fiscal year 2017 to 0.961 Tcf in fiscal year 2019 and is expected to continue falling. Current reserves will provide 0.365 Tcf per year by 2030. Bangladesh might be completely out of natural gas by 2041 if no action is taken [10]. This disease of natural gas depletion has spread across the tables of those responsible for making policy, as the consequences of electricity unavailability in Bangladesh can be demonstrably severe. Unscheduled load shedding can cripple business operations and industrial productivity, thereby hindering economic growth. They also hinder rural development efforts. Furthermore, the quality of life for citizens can be significantly diminished by limited access to services [11]. Because of this, Bangladesh places a lot of importance on making electricity with nuclear energy so that it can become less reliant on natural gas. Fig. 4 depicts the current installed capacity of electricity generation by source.

### 4.2 Future electricity generation scenario

**Fig. 5:**
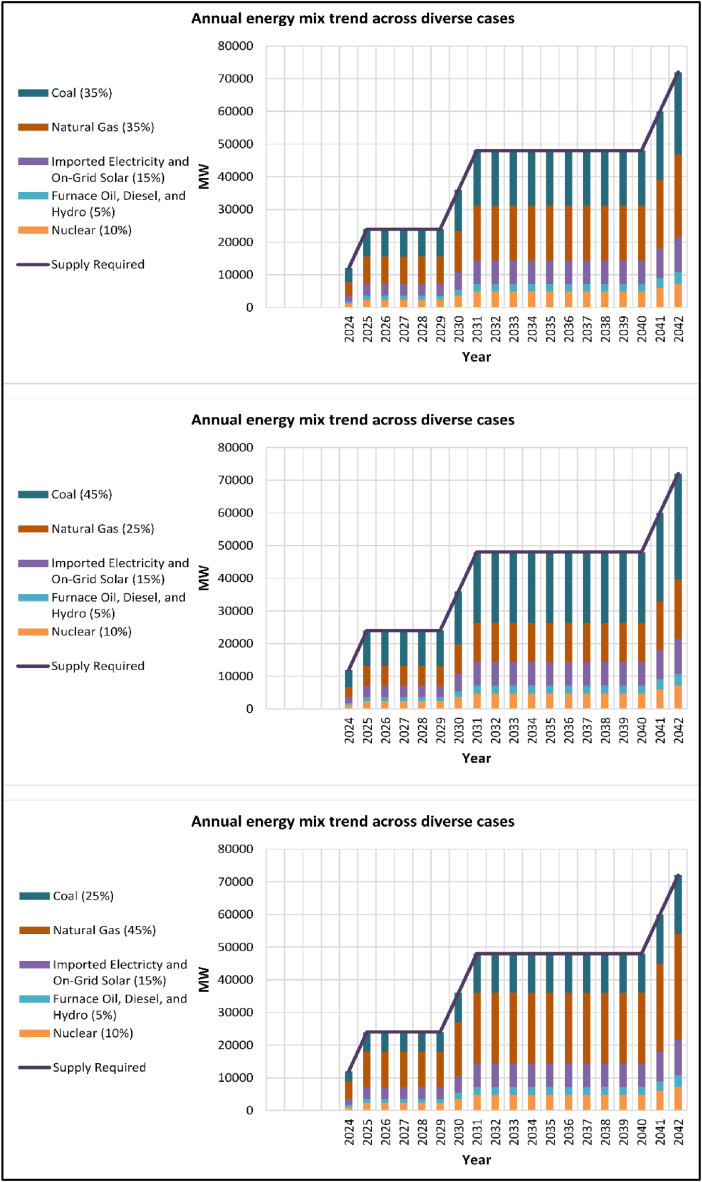
Annual energy mix trend across diverse cases.

In Fig. 5, an analysis was carried out concerning the yearly energy composition trends for various scenarios covering the period from 2024 to 2042 subsequent to the integration of nuclear energy into the portfolio of energy sources. The forecast was established based on the data extracted from PSMP 2016. The predictions suggest that the expected electricity demand will reach approximately 72,000 MW by the year 2042. Within this specific scenario, three distinct situations were taken into account. When comparing the existing installed capacity for electricity generation by source as illustrated in Fig. 4 with the projected annual energy mix as depicted in Fig. 5, nuclear energy is maintained at a consistent 10 percent across all instances due to its substantial initial investment requirement that could potentially strain the economy. Additionally, allocations were put in place for furnace oil, diesel, and hydro energy resources in all scenarios at a rate of 5 percent, with the current distributions fixed at 35 percent. The reduction in reliance on furnace oil and diesel can be mainly linked to the exorbitant expenses associated with the importation of these fuels and the adverse environmental effects they bring about. The geographical landscape of Bangladesh is not conducive to the establishment of hydro based power plants, hence the low allocation for hydro energy. At present, 6 percent of the electricity supply is sourced from on-grid solar installations and imported electricity from neighboring countries. Across all three forecasted scenarios, this percentage is maintained at 15 percent to ensure consistency and stability in the energy mix. Currently, 59 percent of electricity comes from natural gas and coal, and in all three forecasting cases, 70 percent of electricity will come from these sources. Currently, 51 percent of the energy mix is dependent on natural gas. As natural gas sources are depleting, it is considered 35 percent, 45 percent, and 25 percent, respectively, in three cases. In terms of coal, a rise is seen despite reducing dependency on natural gas. Currently, 8 percent of electricity comes from coal, and in forecasted cases, it is considered 35 percent, 45 percent, and 25 percent, respectively.

Here, the most significant shift is anticipated in the reliance on natural gas and coal. While their combined allocation is projected to remain at 70 percent in the projected three cases, the individual percentages vary based on the scenario. This is primarily driven by the existing infrastructure for these sources and the relatively long construction times required for new power plants rather than upgrades. In essence, the forecast outlines a gradual move away from cost-prohibitive and environmentally detrimental fuels like furnace oil and diesel, paving the way for a more secure and sustainable mix that incorporates nuclear, on-grid solar, and imported electricity. However, due to existing infrastructure and reliance, natural gas and coal are expected to remain prominent players in Bangladesh's energy landscape for the foreseeable future.

### 4.3 Balanced approach (coal 35 % and natural gas 35 %)

• Change: Significant increase in coal usage to compensate for the declining natural gas.
• Impact: Meets short term demand and offers some energy security in the near future, but with increased environmental impact and potential long term cost issues (coal import dependence) due to high coal use.

### 4.4 Prioritizing coal (coal 45 % and natural gas 25 %)

• Change: Faster decrease in natural gas, replaced by increased and continued coal use.
• Impact: Coal combustion is one of the major sources of greenhouse gas emissions. Shifting heavily towards coal requires significant investments in new coal based power plants and related infrastructure. This can lock Bangladesh into a high-carbon energy system for decades.

### 4.5 Prioritizing natural gas (coal 25 % and natural gas 45 %)

• Change: Slower decline in natural gas compared to other scenarios, with a moderate increase in coal.
• Impact: Offers some level of energy security in the short to medium term with slower natural gas depletion.

Nuclear energy presents a complex but potentially beneficial addition to Bangladesh's energy mix. By carefully weighing the strengths, weaknesses, opportunities, and threats and considering the justifications behind each scenario, Bangladesh can make informed decisions about integrating nuclear energy while ensuring a secure, reliable, and ultimately sustainable energy future.

## 5 The beginning of the nuclear age

The concept of constructing a nuclear power plant in the northwest of Bangladesh was initially discussed all the way back in 1961. Site selection and land acquisition at Rooppur were done in 1963. Regrettably, it was cancelled in 1970. After Bangladesh became independent, its government started talking to the Soviet Union in 1974, but no deal was made. A proposal for a 125 MW nuclear power plant was authorized in 1980. Unfortunately, even this attempt failed. Another feasibility study was performed in 1987–1988, and it was decided to build a 300–500 MW nuclear power plant. But steps were initiated in 1998 to build a 600 MW nuclear power plant. In the year 2000, the nuclear action plan was given the go-ahead for implementation. In 2009, after a long wait of 48 years, the dream became a reality for the people of Bangladesh. The governments of the People's Republic of Bangladesh and the Russian Federation finally came up with a memorandum of understanding for cooperation on peaceful nuclear energy use. The visionary leadership of the honorable Prime Minister Sheikh Hasina was instrumental in the development of this agreement [12]. In 2011, construction was started by the responsible parties involved in the project. Two VVER-1200 reactors will be installed at Rooppur Nuclear Power Plant. The capacity of the power plant will be 2400 MW. It is expected that one of two units will be commissioned by the last quartile of 2024.

## 6 Methodology: SWOT analysis

A system's performance, quality, and prospects can be evaluated with the help of the SWOT analysis, which considers the system's strengths, weaknesses, opportunities, and threats in order to implement some degree of strategies for management to capitalize on the system's strengths, make the most of its opportunities, mitigate the system's weaknesses, and protect itself from potential threats [13]. The technique is useful for weighing the positive and negative effects of a variety of elements on a system. SWOT analysis for energy planning has been well-recognized and accepted within the academic community. A comprehensive SWOT analysis was conducted to identify the key factors and problems that need to be addressed for the purpose of expanding the energy sector in Tunisia and attracting potential investors to get involved with the sector [14]. Lei et al. used a SWOT analysis to assess the photovoltaic solar power sector in Africa, comparing it with the corresponding sector in China [15]. Olabi et al. conducted comprehensive research on the significance of battery energy storage systems in the context of electricity transmission. They examined the advantages and disadvantages of the systems via a SWOT analysis [16]. A study was conducted to assess the status of renewable energy in Mazowieckie Voivodeship, Poland. This included the implementation of a SWOT analysis and the subsequent estimation of the region's renewable energy potential [17]. Kamran et al. also used the SWOT analysis technique to study the renewable energy sector in Pakistan [18]. The SWOT model has been employed to examine the various growth strategies employed within China's bio-energy sector [19]. Furthermore, SWOT analysis was used to evaluate Nigeria's nuclear exploration plans [20]. There are advantages to this approach to analysis, including its low cost and the fact that anybody familiar with the topic being studied can utilize it effectively.

Our study breaks new ground in analyzing Bangladesh's nuclear energy policy. As the first comprehensive effort of its kind, it stands to offer valuable insights. The study equips policymakers with the knowledge necessary to navigate the complex considerations surrounding nuclear energy. This consequently facilitates the way for informed decision-making that will shape the future of Bangladesh's nuclear energy landscape.

## 7 Result analysis

This section offers an in-depth analysis of various factors that were carefully evaluated in the context of this research, highlighting the thorough examination of different aspects that played a crucial role in shaping the study's framework.

The significant input provided by the government of the People's Republic of Bangladesh to this SWOT analysis has played a crucial and pivotal role in shaping the outcomes and insights derived from this strategic evaluation. They provided a cornerstone of data essential for evaluating the feasibility and potential pitfalls of nuclear energy adoption. This included official policy documents outlining their vision for integrating nuclear energy into the power grid. Additionally, they shared data on the existing transmission and distribution infrastructure and its capacity, allowing the study to assess how nuclear energy could complement and expand Bangladesh's ability to meet its growing electricity demands. Finally, transparency regarding the financing of the construction of a nuclear power plant in Bangladesh was equally imperative. This data helped the study determine the economic viability of the plans, considering not just capital costs but also operation and maintenance costs.

To enrich the analysis further and broaden its perspective, this research also delved into a diverse array of external sources. Studies on worldwide nuclear power plant projects provided valuable insights into best practices, potential challenges encountered in other countries, and various technological advancements. Perspectives from recognized experts in this field, gleaned from reputable news outlets and published scholarly literature, offered valuable insights and considerations not limited to the Bangladesh context.

**Fig. 6:**
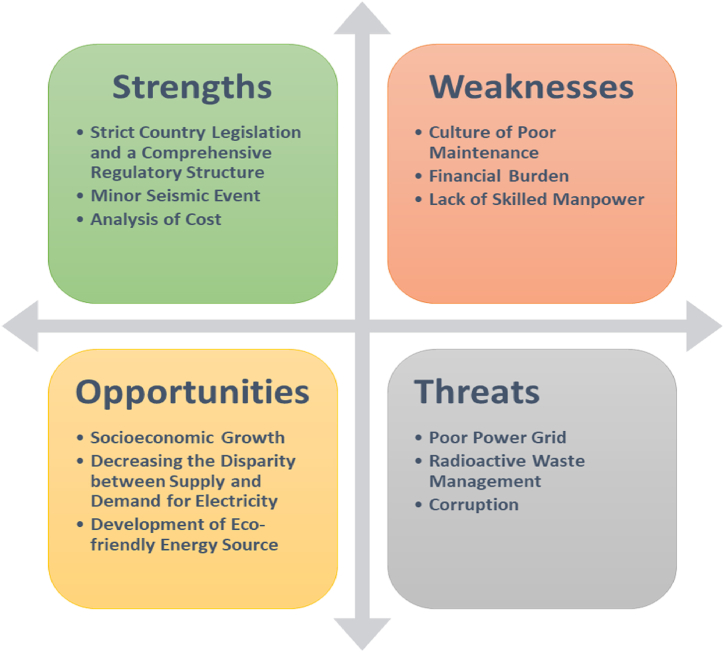
SWOT matrix.

By combining extensive data from the government and a wide range of external sources, the SWOT analysis was able to present a more insightful and realistic picture of Bangladesh's prospects for adopting nuclear energy. It not only identified potential benefits but also highlighted potential issues and areas that require careful consideration. Fig. 6 is about the state of nuclear energy readiness in Bangladesh by using SWOT analysis.

### 7.1 Strengths

#### 7.1.1 Strict country legislation and a comprehensive regulatory structure

Maintaining a high standard of safety at any nuclear site is essential for protecting employees, citizens, and the environment from unnecessary radiation risks. Protecting employees, citizens, and the environment from any potential ill consequences of nuclear site operation is the first priority of any regulatory body. At the current time, the promotion and control of nuclear technology in Bangladesh is both within the purview of the Bangladesh Atomic Energy Commission (BAEC). When it comes to the safe and secure use of nuclear technology for peaceful purposes, BAEC is the ultimate authority to turn to for guidance. BAEC's regulatory arm is the Nuclear Safety and Radiation Control Division (NSRCD). The director of the NSRCD oversees the organization's regulatory efforts throughout the country [21]. In light of its enduring dedication to nuclear energy, BAEC integrates strategies for decommissioning nuclear power plants after the end of their service life, in addition to handling radioactive waste. The proactive approach was showcased in 2015 when BAEC took the lead in forming the Nuclear Power Plant Company Bangladesh Limited (NPCBL), with the specific responsibility of supervising the operational and maintenance activities of the Rooppur Nuclear Power Plant and all future nuclear power plants to be constructed [22]. NPCBL is tasked with the duty of carrying out the decommissioning procedure of nuclear power plants at the end of their operational life while strictly following the safety criteria specified by the IAEA, of which Bangladesh is a committed member [23]. In addition, Russia will provide technical support in this regard as per the memorandum of understanding of 2011 [24]. The forecasted decommissioning outlay for the Rooppur Nuclear Power Plant is estimated to be a considerable USD 527 million, underscoring the detailed planning and financial assessments imperative for the rosperous execution of such crucial proceedings [25].

#### 7.1.2 Minor seismic event

**Fig. 7:**
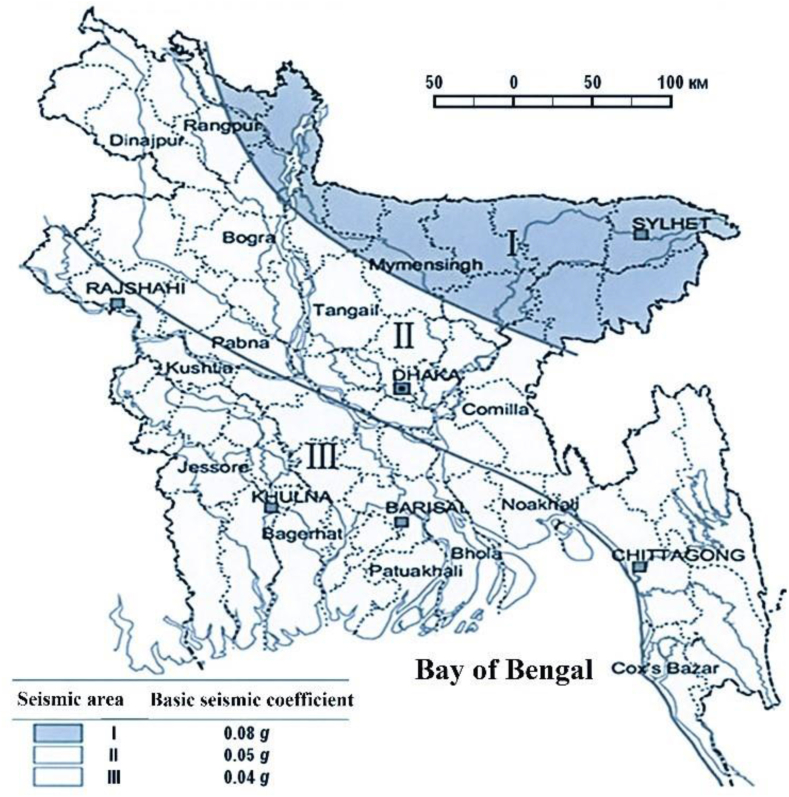
Seismic map of Bangladesh.

Countries with a high population density are more likely to experience devastating seismic events. This calls for a thorough examination of the situation prior to the construction of a nuclear power plant, as per regulations set out by the International Atomic Energy Agency (IAEA). Experts agree that the Bangladesh nuclear power plant and its equipment are safe to operate during seismic events up to eight points on the MSK-64 scale [26]. In Fig. 7, we see a seismic map of Bangladesh drawn to scale using survey data. The area where the Rooppur Nuclear Power Plant would be constructed is classified as seismic zone II, which is rather stable.

#### 7.1.3 Analysis of cost

**Fig. 8:**
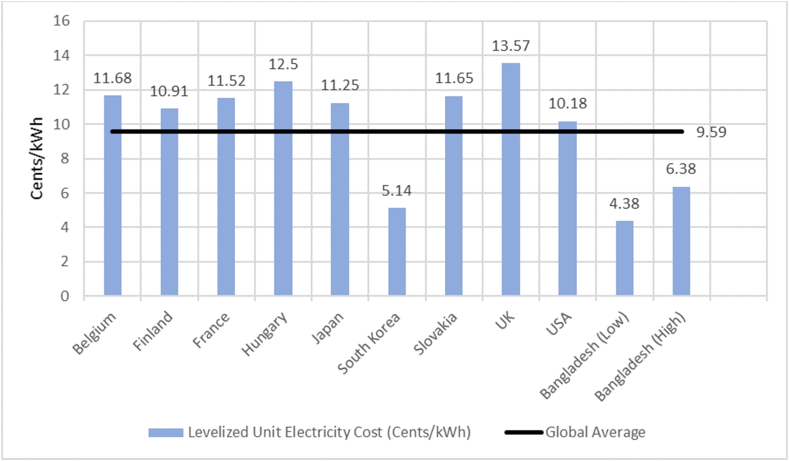
Variations in the global Levelized Unit Electricity Cost [30].

**Fig. 9:**
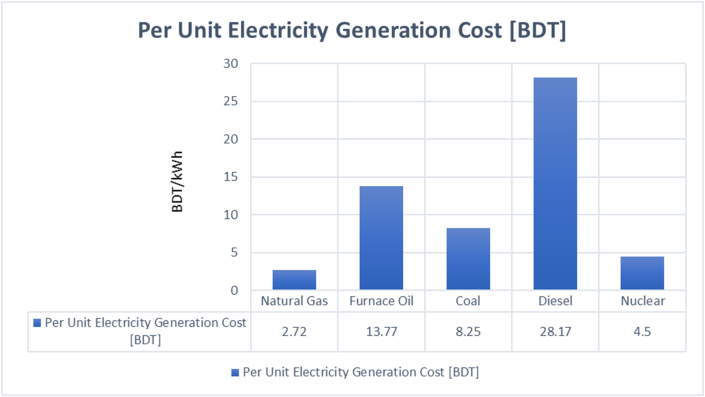
Per unit electricity generation cost of various fuels [27].

As stated earlier, the Rooppur Nuclear Power Plant will have the capacity to generate 2400 MW of electricity. During at least the first two decades, the cost of electricity generation at the Rooppur Nuclear Power Plant will be between BDT 4 and BDT 4.50 per unit, which is substantially lower than the cost of coal-fired plants but more than that of natural gas-fired plants in Bangladesh [27]. In addition, compared to coal-fired power plants, nuclear power plants have lower operating and maintenance costs [28]. According to estimations, the VVER-1200 reactor is designed for a plant capacity factor of 90 percent, while the global average is 85 percent [29]. Islam and Bhuiyan conducted a comprehensive cost assessment of the Rooppur Nuclear Power Plant [30]. Their findings indicate that the plant's operation and maintenance costs range from USD 14.5 to USD 7.82 per MWh, while the fuel costs range from USD 11.2 to USD 4.5 per MWh. These cost estimates take into account a plant capacity factor ranging from 85 percent to 50 percent. Fig. 8 illustrates the global Levelized Unit Electricity Cost associated with nuclear power plants. It illustrates the proximity of Bangladesh to South Korea as well as its comparatively lower unit generating costs, which are around half of the global average. Furthermore, the reactors of the Rooppur Nuclear Power Plant are expected to have a service life of 60 years. Though the initial capital cost of a nuclear power plant is high and might put a strain on the economy of a developing country, it is apparent that Bangladesh would gain from the Rooppur Nuclear Power Plant in the long run. Fig. 9 represents a cost comparison between nuclear and various fuels used for electricity generation in Bangladesh.

### 7.2 Weaknesses

#### 7.2.1 Culture of poor maintenance

A crucial component of quality management projections is maintenance. It is essential for maintaining the functionality and productivity of infrastructure and other priceless assets. Poor maintenance practices have long plagued Bangladesh's power grid, which has been a significant obstacle to the introduction of a nuclear power plant. In the field of nuclear technology, this deplorable culture cannot be tolerated. A country's capability for the integration of a nuclear power plant into its power grid may be determined in large part by looking at its safety culture. It is generally agreed that understanding the dynamics of safety principles is a useful thing for changing people's actions in order to significantly boost overall safety performance [31]. Since the smallest mistake may have fatal consequences, the site of a nuclear power plant needs the greatest level of safety and prudence. In order for Bangladesh to achieve its goal of incorporating nuclear power plants into its power grid, it is crucial that the country deliberately improves upon its maintenance culture.

#### 7.2.2 Financial burden

A nuclear power plant has a number of characteristics, including a high capital cost that makes it more sensitive to interest rates from development agencies. It is typical for a nuclear power plant to not use a fixed-cost concept. In the USA, France, and other industrialized countries, it is obvious. Every nuclear power plant constructed in the USA between 1966 and 1976 saw cost increases of 300 percent on average. The project cost has already gone up at the Rooppur Nuclear Power Plant. The 2018–19 Annual Development Programme (ADB) would allocate around USD 13.08 billion, which is USD 1.1 billion more than the previous year. USD 12.65 billion was the prior cost projection. Russia will lend USD 11.38 billion, or 90 percent of the total anticipated cost. Bangladesh has to pay back the debt with a 10-year grace period in 28 years. The interest rate is 1.75 percent plus the London interbank offered rate, which cannot exceed 4 percent. Even if the project cost stays the same, it would still cost Bangladesh at least USD 20 billion [32]. And, obviously, when additional expenditures are taken into consideration, this project's cost will be higher. For instance, in 2017, the Executive Committee of the National Economic Council (ECNEC) authorized a BDT 956 crore project of river dredging in the Padma for the transportation needs of the nuclear power plant [33]. When everything is considered, it is not at all budget-friendly as it requires high capital costs, though operation and maintenance costs are low as stated earlier. Therefore, it is essential to have a wide-ranging public debate on the establishment of nuclear power plants in order to reach a single, binding national decision.

#### 7.2.3 Lack of skilled manpower

The biggest problem with the operation of Rooppur Nuclear Power Plant is that Bangladesh doesn't have enough engineers who are skilled and experienced in nuclear technology. Officials with the project say that Bangladesh would have to depend on Russian experts during the first few years of plant operation. Bangladesh is currently constructing its first nuclear power plant, which will start operating in 2024. However, the number of universities in the country offering nuclear engineering or nuclear science programs is limited. Master's, M.Phil., and Ph.D. programs are currently available at the University of Dhaka, Jahangirnagar University, University of Rajshahi, University of Chittagong, Shahjalal University of Science and Technology, and Bangladesh University of Engineering and Technology, but each intake a small number of students due to resource shortages [34]. This lack of nuclear-related education opportunities in Bangladesh makes it hard to find and train a large number of skilled people needed to run a nuclear power plant safely and well. Furthermore, Bangladesh has a relatively small skilled population, which may make it more difficult to find highly skilled engineers and technicians with experience in nuclear technology. Additionally, the high level of technical expertise and knowledge required to operate a nuclear power plant safely and efficiently is difficult to acquire without proper training and education. All these constraints make it a challenging task for Bangladesh to find the necessary skilled manpower to operate a nuclear power plant safely and efficiently.

### 7.3 Opportunities

#### 7.3.1 Socioeconomic growth

The Rooppur Nuclear Power Plant will not only meet the country's need for electricity, but it will also help the country's economy and society grow as a whole. There is already an Export Processing Zone (EPZ) in Ishwardi, which spans a total area of 309 acres and has 324 industrial plots. When the Rooppur Nuclear Power Plant becomes operational, Ishwardi EPZ will have unrestricted access to electricity [35]. Since it encourages growth in the country's industrial sector, it will help the economy as a whole. It will help the local economy by creating new jobs and giving the government more tax money. The project site also employs over 3000 foreigners. The local economy has greatly benefited from these foreigners. A large number of foreigners has resulted in the opening of several new retail centers by local people, showing the emergence of small enterprises. The Rooppur Nuclear Power Plant will serve as a catalyst for the growth of the regional economy and infrastructure. One such piece of ancillary infrastructure constructed for the Rooppur Nuclear Power Plant is the Padma River Port. The project site will also be connected by a railroad that will be constructed.

#### 7.3.2 Decreasing the disparity between supply and demand for electricity

Enhanced economic activities in Bangladesh, together with continued GDP growth, are driving up electricity demand at a rapid rate. Bangladesh will need an extra 34,000 MW of electricity by 2030 if its GDP grows by more than 8 percent per year [36]. The demand for electricity is growing at a rate of 9–10 percent annually and is forecast to keep rising in the years ahead [37].

Rooppur Nuclear Power Plant is projected to generate 19.34 billion kilowatt hours (kWh) of electricity per year, enough to meet the annual electrical needs of 60 million people. It will also be useful in helping to level the playing field between the country's eastern and western regions in terms of electricity generation and consumption. Numerous factories will be constructed if there is an adequate and reliable electricity supply.

#### 7.3.3 Development of eco-friendly energy source

**Fig. 10:**
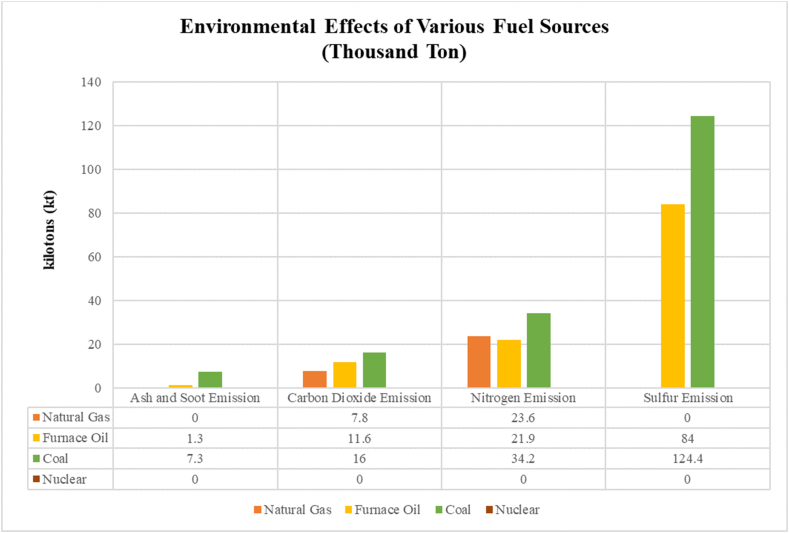
Environmental effects of various fuel sources [39].

Driven by the finite availability of fossil fuels and their detrimental environmental impact, a worldwide shift towards a variety of energy sources is underway. Nuclear energy emerges as a promising alternative in this pursuit of eco-friendly solutions for the looming environmental issue. To achieve a sustainable and environmentally sound energy structure, Ahmed et al. [38] recommended a collaborative approach. Their strategy integrates effective energy policies for all sources, fostering long-term development. Nuclear energy's energy density and internalization of health and environmental costs provide it with a strong competitive position from the standpoint of sustainable development. The main benefit of nuclear power plants is that they don't need oxygen to generate electricity, and they don't emit any pollutants that affect the environment. The environmental impacts of running a 1 GW electricity energy block for a year using various fuel sources are shown in Fig. 10.

### 7.4 Threats

#### 7.4.1 Poor power grid

**Fig. 11:**
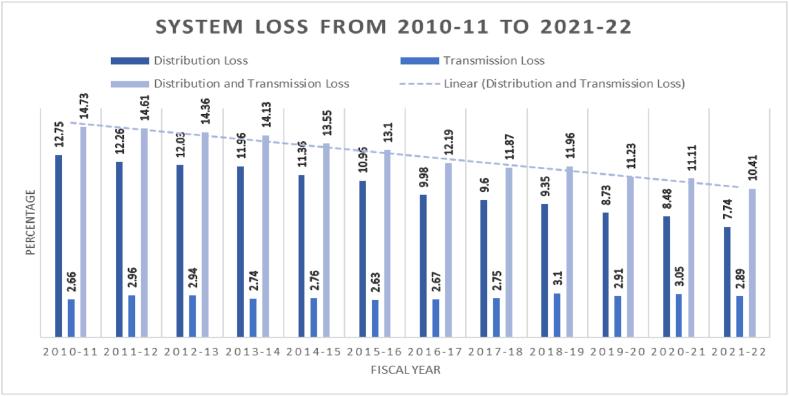
Distribution and transmission loss in Bangladesh [40].

The outdated power grid in Bangladesh is a significant issue. As many transmission and distribution lines were established decades ago, electricity outages and system losses occur often. In addition, the grid is poorly linked, leaving many rural communities unconnected. Despite these challenges, there are efforts underway to improve the power grid and make it more capable of supporting nuclear power plants. The government has announced plans to upgrade the transmission and distribution infrastructure, as well as to connect more rural areas to the grid. However, these efforts will take time and significant investment to implement. Fig. 11 displays the present trend of one of the key performance indicators, i.e., system losses, from the fiscal year 2010-11 through the fiscal year 2021–2022. The system loss has decreased from 14.73 percent in the fiscal year 2010–2011 to 10.41 percent in the fiscal year 2021-22.

#### 7.4.2 Radioactive waste management

The successful deployment of nuclear energy for electricity generation is contingent upon the implementation of robust radioactive waste management strategies. The absence of such strategies can foster public apprehension and skew public perception of nuclear energy's overall risk-benefit profile [41]. Despite the fact that spent fuel from the Rooppur Nuclear Power Plant is slated to be brought back to Russia through a separate agreement. Bangladesh is responsible for the treatment, storage, and transportation of low and intermediate level waste within the country. As well, a decision regarding the disposal site and options for low and intermediate level waste is pending. This includes surface, near-surface, underground, etc. [42]. It is critical to note that the safety and security of low and intermediate level waste is paramount. The final decision must meet the most stringent standards of safety and environmental protection.

#### 7.4.3 Corruption

The misuse of trusted authority for the sake of personal gain is one definition of corruption [43]. Anti-corruption efforts in Bangladesh have been ongoing. According to Transparency International's 2017 Corruption Perceptions Index (CPI), Bangladesh scored 28 out of 100 points, placing it at position 143 out of 180 countries [44]. These findings do not inspire confidence that a large investment, such as a nuclear power plant, would go off without a hitch. It terrifies investors from all sectors, and the battle against it should not be relegated to the government; it should be the duty of all people.

## 8 Discussion

This article examines Bangladesh's nuclear energy strategy in terms of its strengths, weaknesses, opportunities, and threats. With data retrieved from PSMP 2016, it also explores multiple possibilities for the future energy mix. Bangladesh's entry into nuclear energy presents a multifaceted endeavor with the potential for significant transformation. This has made it evident that Bangladesh has made the courageous decision to become energy self-sufficient, which will help Bangladesh foster socioeconomic growth and GDP. This is a remarkable step that is essential to the country's development and will ensure a bright future for Bangladesh. Bangladesh's nuclear energy strategy will test the country's commitment to sustainable development. Bangladesh needs to meet all safety standards and safeguard the environment from radioactive waste. This will require a strong commitment from government, industry, and citizens alike. Furthermore, Bangladesh must invest in skilled manpower to ensure nuclear technology is safe and secure. By hiring skilled personnel, we can prevent poor maintenance practices. This endeavor not only strengthens the field of nuclear energy but also infuses valuable technical expertise into the broader societal framework. The potential of nuclear energy is undeniable, but public trust must be built for its successful implementation. Transparency and open communication are essential in this case. The government needs to clear public perceptions regarding financing, safety measures, and radioactive waste management. By providing clear and accessible information, the government can dispel rumors and build public confidence in the strategy. Our research acknowledges this gap in understanding public perception, highlighting the need for future surveys to gauge public anxieties and tailor communication strategies accordingly.

## 9 Conclusion

For Bangladesh, the fact that nuclear energy has the potential to make a sustainable and long-term contribution to the country's overall macroeconomic development is something that has the potential to be beneficial to a variety of different sectors, including the energy sector as well as the industrial sector. Even though the SWOT analysis showed that Bangladesh's journey to adopt nuclear energy has some weaknesses and threats, we can say that it is in a strong position in terms of its strengths and opportunities as the weaknesses and threats can be resolved over time by taking sufficient action plans and the government of the People's Republic of Bangladesh is working on it. A number of steps have already been taken, for instance, the Bangladesh Atomic Energy Commission Act, which was passed in 2017. The country is also in the process of developing its power grid. The power grid network strengthening project is ongoing under the Power Grid Company of Bangladesh Limited (PGCB). To combat threats such as corruption, effective combined measures are required, and these issues are generally associated with emerging economies. Bangladesh ought to construct more nuclear power plants. Although it does not provide a miracle fix, nuclear energy can help keep up with the rising demand for electricity. The article can also benefit emerging economies that intend to construct nuclear power plants in the near future.

## Data availability statement

Data will be made available upon request.

## CRediT authorship contribution statement

**Faiyaz Fahim:** Writing – review & editing, Project administration, Methodology, Formal analysis, Data curation, Conceptualization. **Abdulla Al Farabi:** Writing – original draft, Resources, Investigation. **Md Sabid Hasan:** Writing – original draft, Visualization, Resources, Formal analysis, Data curation. **S.M. Naimur Rhaman Sayam:** Writing – review & editing, Writing – original draft, Visualization.

## Declaration of competing interest

The authors declare that they have no known competing financial interests or personal relationships that could have appeared to influence the work reported in this paper.
